# Silver and X-Rays in Diseases of the Alimentary Tract

**Published:** 1912-02

**Authors:** 


SILVER AND X-RAYS IN DISEASES OF THE ALIMENTARY TRACT (F. Hernaman-Johnson: *British Med. Jour.,* October 14, 1911).—Acting upon the suggestion made last year by Sir J. J. Thomson as to the use of secondary rays in therapeutics (see PRESCRIBER, 1910, p. 184), the author concluded that if silver, in a suitable form, could be introduced into the alimentary tract and made to cover an area of ulceration, the result of exciting the metal by hard *x*-rays projected through the back of the ulcer would be a bombardment of the diseased surface similar in effect to that from a highly active radium salt. He employs chemically pure precipitated silver, which is a dark, amorphous, tasteless powder, giving 8 grains of this mixed intimately with a meal of milk and breadcrumbs. Seven hours later an exposure was made (full details of technic are given), and this procedure was repeated three times a week for several weeks. The method has been applied with considerable success to cases of duodenal and gastric ulcer and to a case of pyloric obstruction with tumour. The author suggests, in place of administering silver by the mouth, the injection (per rectum or subcutaneously) of an emulsion of precipitated silver in olive oil.

(Note. We have used the high frequency current with the hope of driving silver from solutions of pretargol, etc., into rectal ulcer and cancer—apparently with good results though the time is not ripe for a definite report. A. L. B.)

